# Mother-infant consultation during drug treatment: Research and innovative clinical practice

**DOI:** 10.1186/1477-7517-5-6

**Published:** 2008-02-06

**Authors:** CF Zachariah Boukydis, Barry M Lester

**Affiliations:** 1 Erikson Institute, 420 N. Wabash Ave., Chicago, Illinois 60611-5627, USA; 2 Brown Center for the Study of Children at Risk, Women and Infants Hospital, 101 Dudley Street, Providence, RI 02905, USA

## Abstract

**Background:**

This paper details a model for consulting with mothers and infants, and drug treatment staff used in a residential drug treatment program and relevant to other treatment settings. The role of parent-infant consultation based on the Neonatal Network Neurobehavioral Scale (NNNS) was evaluated.

**Methods:**

A sequential cohort model was used to assign participants to 1. NNNS consultation versus 2. standard care. The effects of NNNS consultation were evaluated using the Parenting Stress Index and NNNS summary scores.

**Results:**

Participants in the NNNS consultation condition had significantly less stress overall, and less stress related to infant behavior than participants in standard care. There were no differences in infant behavior on the NNNS Summary scores.

**Conclusion:**

The implications for NNNS consultation in drug treatment programs is outlined. The importance of prevention/intervention to establish satisfactory mother-infant interaction in recovery programs which include a central parenting component is indicated.

## Introduction

In the past fifteen year, there have been marked changes in drug treatment services for women (Finkelstein, 1996 [1]; Homan et al, 1993[2]; Clayson, Berkowitz & Brindis, 1995[3]; Lester, Twomey, Boukydis, 2000[4]). One central feature to these services is the recognition of challenges that many women of childbearing age face to progress in recovery, and as mothers, to grow and mature as parents with their children. There is an identified need to integrate parenting support and education into traditional drug treatment programs (Weissman et al, 1995[5]; Jones, 2006 [6]). Programs which combine drug treatment and parenting services are more likely to retain women in treatment and decrease the likelihood of relapse (Roberts & Nishimoto, 1996[7]; Szuster et al, 1996[8]; Kaltenbach & Finnegan, 1998[9]; Jones, 2006[6]). There has also been a need to integrate and evaluate new models derived from fields such as child development, applied developmental psychology and infant mental health (Lester, Affleck. Boukydis, Freier & Boris, 1996[10]; Sameroff, 2004[11]). The central focus of this paper is on the use of neonatal assessment to consult with mothers and infants in order to improve maternal ability to read the unique signals of their infant, provide a satisfactory beginning to early parenting and complement steps in recovery.

### Interaction between drug abusing women and their infants

There is wide variation in the neurobehavioral effects of substance use on the infant (Lester et al, 2002[12]). Reviews of neurobehavioral development of substance exposed infants point to the most common findings of problems with regulation of states of arousal, irritability, and challenges to motor control (Lester et al, 2002 [12]). These factors can make infants difficult to read, and manage. Therefore, the infant's behavior may affect the mother's ability to help the infant to regulate states of arousal. Many studies of mother-infant interaction in the population of substance using women and their infants indicate problems in mother-infant interaction – particularly reading infant's signals, effective soothing and management strategies and successful management of daily cycles of feeding, sleep, and play (Kaltenbach & Finegan, 1998 [9]). These early difficulties can lead to increasing parental stress, maternal reactivity, lowered maternal self esteem, difficulties in arranging the environment to meet the infant's needs for appropriate stimulation, and difficulties in the early formation of the attachment relationship between mother and infant (Egeland & Erikson, 1990 [13]). The perinatal period is a critical time for supporting women as they take on the responsibilities of parenting while still learning the emotional lessons necessary for themselves in treatment and recovery (Ewing, 1992 [14]; Clay, 1997 [15]).

To the extent that the parenting component of treatment programs address and ameliorate these difficulties, there should be a change in women's ability to interact with, and manage their infants. In addition, there should also be a reduction in parenting stress and reactivity, and mothers should be more able to effectively organize the caregiving environment to meet their infant's needs. As drug abusing women participate in parenting services while in treatment with their infants, they use individual sessions to enhance learning and/or remove barriers to understanding and managing the infant; and work in treatment on issues touched off by their reaction to their infant and their emerging identity as a mother.(Janson et al, 1996 [16]; Jones, 2006 [6]). Thus, the individual consultation model detailed in this paper operates at two levels: 1. Observing and articulating the meaning of infant behavior; and 2. Maternal observations and changing maternal misperceptions (Zeanah, Benoit, Hirschberg, Barton & Regan, 1994 [17]) of their infant behavior.

### Clinical rationale for newborn/early infancy consultation in treatment settings

After an infant is born, there is an opportunity to support the mother's early attachment to her infant while consultant and mother 'observe together' (Clark, Tluczek and Gallagher, 2004[18]) to see how the infant is functioning. The time after an infant is born is particularly important because it is a time of rapid change in the mother's self concept, adjustment to the newborn, and potential availability for being a participant in a secure attachment relationship (Sameroff, 2004[11]). If the relationship between mother and infant is able to 'stabilize' into mutually satisfying experiences, there is the potential for reinforcing more intrinsic ability on the mother's part to read and respond to processes of change as the infant develops (Papousek and Papousek, 1987[19]). The mother's emerging self awareness and self control in treatment can be paralleled by her awareness of her ability to soothe her infant and to help her infant continue to develop self control. There is also the potential for the mother to strengthen or recapture her parenting capability in the face of a history of being parented inconsistently. The mother's own parenting history may provide excessive challenges to her ability to empathize with her infant's needs.

The NNNS consultation as it has evolved in training and practice, is collaborative between the mother and the consultant, and does not involve an expert driven demonstration of the infant's behavior and functioning. The authors have extensive experience using neonatal assessment to consult with parents of at-risk infants (Boukydis & Lester, 1999[20]; Boukydis et al 2004[21]) and women in residential and day treatment programs. Over time this work has extended to training parenting consultants from different disciplines, including drug treatment staff themselves to consult with women and their infants. In the work reported in this paper, the Neonatal Network Neurobehavioral Scale (NNNS); Lester & Tronick, 2001[22], 2004[23]; Boukydis & Lester, 1999[20]) was used in the first two months after infants were born to provide a context for collaborative consultation.

The NNNS was developed for use in the Maternal Lifestyles Study (MLS) a federally collaborative multi-site study funded by NIDA and NIH (Lester et al, 2002[12]). The NNNS is a neurobehavioral assessment of at-risk (substance exposed, preterm infants) used in research and clinical settings (Boukydis & Lester, 1999 [20]; Boukydis, Bigsby and Lester, 2004 [21]; Bigsby, Boukydis, Andreozzi and Lester, 2004[24]) as well as structured context for consulting with parents and caregivers about the behavior and needs of at-risk infants. The NNNS is particularly useful in this work because it was developed to be sensitive as an assessment for at-risk drug exposed infants and has been validated in large multi-site studies of drug-exposed infants cocaine, heroin, alcohol, methamphetatime, polydrug) and their caregivers.

In general, the NNNS shares the same basic underlying philosophy or framework of behavioral organization of the infant common to a family of neonatal assessments: the NBAS for full-term infants (Brazelton and Nugent, 1995[25]) ; the APIB for preterm infants (Als et al, 1982[26]) and the NNNS for substance-exposed and preterm infants (Lester & Tronick, 2001 [23], 2004 [24]). There has been extensive research done using these neonatal assessments to consult with parents of at risk infants (Meyer et al, 1994[27] Das Eiden & Reiffman, 1996[28]). Previous work with at-risk preterm infants, using neurobehavioral consultation as part of a comprehensive pre discharge intervention for parents of preterm at -risk infants in the NICU indicated that women who received the type of consultation interacted more effectively in feeding interactions following discharge of the infant from hospital, were less depressed, and had more maternal self confidence than women who received standard care which did not include the neurobehavioral consultation (Meyer et al, 1994[28])

In drug treatment programs with women, the NNNS has been used to consult with mothers and caregivers. The primary purpose for this consultation is to facilitate maternal understanding of her infant; clarify misperceptions or negative 'representations' and prevent disorders of attachment (Zeanah and Boris, 2000[29]).

### Types of consultation with women and substance exposed infants

There are several types of consultation or 'ports of entry' (Sameroff, 2004[11]; Stern, 2004[30]) for the NNNS consultation in drug treatment settings:

#### 1. Complete assessment to highlight different aspects of infant functioning

The purpose of this consultation is to demonstrate a wide range of the infant's behavior and functioning. In this way, it is possible to use the complete NNNS. The consultant comments on the infant's behavior, and behavioral function of the behavior, while performing the NNNS with the mother. The NNNS was designed to elicit a range of behavior and also to elicit the full range of states from the infant during the conduct of the assessment. Thus, it is also possible to examine, and elaborate these areas which are particularly important for mothers and caregivers. The areas include the infant's: relative stability or instability in states, overall irritability, response to handling, ability to be soothed, signs of stress, response to particular handling techniques; responses to animate & inanimate, auditory & visual stimulation and self soothing ability.

#### 2. Partial assessment which highlights different aspects of the infant's functioning

At times, it may be necessary to have a series of consults with caregivers on a regularly scheduled basis. With at-risk infants, stamina and ability to tolerate a lot of handling may be an issue of concern, so it may be necessary to do one or more partial assessments focusing on different 'packages' (a package consists of several interrelated items within the NNNS; some packages focus on motor behavior or reflexes; other packages focus on attention to visual and auditory input). Also, parents may be only able to take in, and observe, a limited range of input about their infant's behavior. Some intervention programs based on the NNNS have delineated different sessions which involve learning about the infant's behavior in different areas of functioning. In the treatment setting, the individual consultation may be paralleled with individual and group sessions where women work on issues touched off by interacting with their infant, and also receive non-judgmental feedback about their handling and parenting capability.

#### 3. Using the NNNS to structure clinical consults for feeding issues and other caretaking tasks

The different areas of functioning in the NNNS can be used to structure consults related to the infant's neurobehavioral functioning. For instance, take the example of a substance-exposed infant who has passed through withdrawal and is just beginning to sustain bottle feeding. Rather than focus on the motor behavior of sucking and swallowing alone, all the NNNS dimensions can be used to focus on the different levels of the infant's functioning and how these may be related to functional feeding. So, the issues of physiological control of breathing, color, oxygenation, are combined with the infant's ability or difficulty in maintaining an appropriate alert state during feeding, the number of signs of stress, the motor control of sucking and swallowing, and the parent's facilitation or possible disruption of the infant's ability to maintain a stable state, initiate pauses in sucking, as well as the parent's ability to recognize and manage signs of stress and availability during the feed. Separate from the feed, the NNNS may be used to consult with the parent about the infant's behavior, and draw implications for how this may relate to feeding. The Clinical Summary (Boukydis, Bigsby and Lester, 2004[22]) indicates dimensions which can be used to reinforce parental observation of the infant's behavior and cues.

#### 4. Consultation related to parental issues; parental anxiety about infant behavior, parental misreading of infant signals

The first two categories of consultation focus primarily on parents observing and articulating infant behavior with the clinician. The third category implies using the NNNS to provide more information related to the parent's management of particular caretaking issues such as feeding. Depending on the issue, the NNNS can be used more interactively, for instance while the parent is handling the infant and an "out loud"observation or 'running commentary' of the infant's state and interactive cues is done by the consultant. Many consults have to do with parental anxiety and how this may make it difficult for a parent to manage their infant's state, or respond effectively to their infant's cues. One example is a parent who talks too loudly, or who talks, makes visual demands, and jiggles, their infant – who may be particularly sensitive or easily overloaded to this sensory input. The infant may avert visually or change state in order to attempt to attain relative homeostatic balance, but the parent takes this averting behavior "personally" and feels that they are being an ineffective parent, or that their infant does not like them. A shared observation of what this infant is doing related to state control would be useful in this situation, as well as possibly discussing, having the parent try, or modeling different strategies to help the infant maintain a stable state, and visual alertness. This could include reducing the intensity of stimulation (i.e. talking more softly) and reducing the number of channels of communication (i.e. looking at the infant, while remaining silent, and sitting steadily holding the infant, without rocking or jiggling the infant). Depending on the relationship with the mother, videotaping of these interactions can be useful for the mother to 'step out of the interaction' and make changes based on her observations and feelings about the interaction (Bernstein & Hans, 1994[31]; McDonough, 2004)[32].

With women in recovery from substance abuse there is often an extreme sensitivity to being told what to do in caring for their infant. Yet, quite often there is a desire to learn satisfying ways to be connected to, take pride in, and learn the unique personal characteristics of their infant. One primary principle of the NNNS consultation involves ways to turn what could be a didactic session into a mutual observation and articulation of the infant's behavioral functioning by wondering aloud, and keeping the focus on the infant's behavior and potential needs. The NNNS Consultation: Feedback for Parents sheet (Appendix 1) indicates how each session can be summarized for feedback to: (a.) the mother; as well as (b.) drug counselors; and (c.) nursing or medical supervisors.

Evaluation of NNNS Consultation.

## Methods

The research involves the evaluation of the NNNS consultation in a residential drug treatment program where women typically entered treatment either in the last trimester of pregnancy or were reunited with their infant during the first month after birth. In the residential program, all women participated in both drug treatment, parenting oriented services, case management, medical care; job training; life management skills; housing assistance and extensive post discharge follow through.

The NNNS Consultation involved women who were engaged in treatment during the last trimester of pregnancy and who remained in treatment after their infant was born. The NNNS Consultation was comprised of two sessions per week during the first month and one session a week for the next month. The overview sequence involved (a.). Introduction, establishing rapport; reviewing infant's behavior in five levels: 1. Physiological; 2. Motor Control/Motor Coordination; 3. State/State Control/Self Soothing; 4. Signs of Stress/Signs of Availability and 5. Capacity for Interaction (Boukydis, 2008[33]); (b.). Managing Feeding and Soothing; responding to particular management issues generated from first consult; (c.). Update on Infant's response to input, handing and soothing; (d.). Managing Infant's Day – consultation on daily patterns of sleep/wake/feed/play; (e.). Continued Observation of infant's development, renewed emphasis on unique preferences for interaction; emerging emotional development; ability to anticipate changes in handling.

The evaluation consisted of analysis of group data from sequential cohorts of women who entered the program during the last trimester of pregnancy. Three consecutive admissions were assigned to the NNNS Consultation group, the next three admissions were assigned to the Standard Treatment group and then the cycle was repeated. Mothers in the Standard Treatment group did not observe the NNNS with their infant. For the purpose of data analysis, one group (NNNS Consultation; NC; N = 16) received the NNNS consultation and the second group (Standard Treatment; ST; N = 15) received full services except for the NNNS consultation. In the Standard Treatment group, the NNNS was done on their infant as a necessary screening assessment. Treatment providers were not aware of group identity and saw the use of the NNNS only as an early screen of infant neurobehavior.

## Results

Basic demographic characteristics of the women and their infants are indicated in Table 1.

**Table 1:** Demographic Variables

**Demographic Variable**	**NNNS Consultation **(N = 16)	**Standard Treatment **(N = 15)
Maternal Age (years)	X = 27.9 yrs	X = 28.2 yrs
Maternal Education	10^th ^grade completed	10.2 grade completed
Infant birthweight (gms.)	X = 2720 gms.	X = 2630 gms.
Gestational age at birth (wks.)	X = 37.6 wks.	X = 37.9 wks.
Ethnicity – African American	42%	45%
Caucasian	28%	30%
Other	30%	25%
Drug Use in Pregnancy		
Cocaine Only	35%	28%
Cocaine/Heroin	15%	20%
Cocaine/Alcohol	50%	52%

Overall ethnicity for the both groups was (42% African American; 36% Caucasian; 18% Hispanic). The two groups did not differ significantly in terms of ethnic composition.

### Parenting Stress

Both groups of women completed the Parenting Stress Index (PSI; Abidin, 1990[34]) when their infant was 2–3 months gestational age. Overall, there were moderately high average levels of parenting stress in both groups total, compared with published findings for other populations of parents (sample X = 143). The Standard Treatment group (N = 15) had significantly higher scores overall (X = 151) than did the NNNS Consultation group (n = 16) of women (X = 134; P <.05). Women in the Standard Treatment group had higher scores on the Stress from Dysfunctional Parent-Child Interaction subscale of the PSI than did women in the NNNS Consultation group. It is possible that women in the NNNS Consultation group were better able to read their infants signals, were better able to help them soothe and experienced less stress from being able to manage their infant's crying and daily patterns of sleeping, feeding and crying.

The NNNS Summary scores at five days for both groups of infants were similar, and were not significantly different between both groups.

## Discussion

Women in the NNNS Consultation group reported less parenting stress, especially for the 'dysfunctional parent-child interaction' dimension than did women in the Standard Care group. The screening assessment with the NNNS did not indicate differences between the two groups based on the NNNS summary scale scores, so it was not likely that differences in group differences in parenting stress were attributable to infant behavior alone. Differences were more likely attributable to differences in maternal ability to read and manage infant states, to soothe infants and to promote infant ability to self soothe. This finding is similar to the findings in our discharge from NICU intervention working using an identical consultation (Meyer et al, 1994 [27]).

The data for this evaluation were limited and there is a need for other types of information, which could enable the examination of the development of mother-infant interactions and infant behavior in both types of groups over time. The use of the NNNS consultation is currently being examined in another protocol with substance using HIV positive women and their infants.

### Training in NNNS consultation

Training in NNNS consultation is be done on two levels: 1. Training of NNNS consultants from different disciplines; and 2. Collateral training of drug treatment staff and other caregivers to observe and articulate infant behavior and development (Boukydis, 2008[33]).

Learning to use the NNNS begins with learning to observe and articulate behavioral observations of infants while lying at rest and their response to being handled during routine care giving activities. A training handout and video observation program have been developed which serve as initial training for NNNS consultants and for drug treatment staff to learn more about observing infants in their programs. It is the ability to observe and articulate infant behavior, which forms the basis for using the NNNS to consult with parents and caregivers. Being able to 'put words onto' what is observed is often useful for parents, who are learning to understand their infant's behavior, states, and state transitions.

After developing the capacity to observe, and organize observations with the behavioral observation framework, the training proceeds to learning a structured assessment of the infant in a way which follows a sequence which is part of the NNNS assessment (Lester and Tronick, 2004[22]). Next the training involves doing short consultations with stable infants and their mothers with the trainer and other trainees observing. Finally the training involves learning to change the type of consultation based on what the infant presents in terms of neurobehavioral organization, how the mother reads and responds to the infants and what the mother's explicit or implicit issues are.

As indicated, the type, and length of consultation may vary, depending on the consultants ability to read the needs of the parents, or conscious agreement between parent and consultant about what the parent hopes to achieve by participating in the consultation. The simplest type of consultation may be 'looking together' at the infant and articulating what is observed – often alternating between parent and consultant in what is observed, and spoken about. This joint observation may occur while the parent, consultant or both are handling the infant.

### Consultation with drug treatment staff

Many providers typically have less formal training in child development and parent-child consultation. As indicated, the importance of the NNNS consultation is that the underlying philosophy of infant observation can be taught to drug treatment personnel with a series of 5–7 training sessions and short-term integrative supervision (Boukydis, 2008 [33]). As providers are more able to observe and articulate the behavior of infant, they are able to see what the infant contributes to mother-infant interaction and caretaking. They are less likely to operate from their own misperceptions about the behavior of drug-exposed infants. They are able to differentiate adaptive strengths in the infant's functioning as well as see where there are areas of concern. They become more effective in observing the follow through on NNNS consultation, and in detailing concerns about the mothers efforts to manage her infants needs and caretaking environment.

## Conclusion

The use of the NNNS consultation has important implications for helping to develop secure attachment relationships between at-risk prenatally substance exposed infants and their mothers or caregivers. In this way the consultation has the promise of preventing future parenting problems. It is also an intervention to address the repair of problematic interactions and misperceptions based on early maternal or caregiver reactions to the drug-exposed infant. The training implies giving drug treatment staff a basic frame of reference based on sophisticated observation of at-risk infant behavior and early interactions, so that they may collaborate with, or incorporate the NNNS consultation into their treatment plans with women in recovery and early stages of responsive/responsible parenting.

## Competing interests

The author(s) declare that they have no competing interests.

## Appendix 1

NNNS Consultation Sheet: Feedback for Parents.

*This sheet can be used to provide a written summary for parents; or to guide feedback to parents when summarizing an NNNS which parents observed*.

1. State/State Changes

a. Summarize the number and type of state changes seen during the consult

b. Describe what events, types of handling, and infant physical movement cause the infant to change states

2. Crying/Soothing

a. Describe when the infant cried or fussed during the consult

b. Describe what the consultant did to soothe the infant.

c. Summary of soothing techniques. Describe which methods of soothing were most effective in helping the infant to achieve a state 4 or lower

3. Infant's Self Soothing/Regulatory Behavior

a. Describe hand to mouth, visual fixation, leg crossing, foot bracing and the changes of state which occurred when the infant performed these behaviors

b. Summarize of self soothing/regulatory behavior seen during the consult

4. Infant's Response to Visual and Auditory Input from Consultant and Parent

a. Describe the infant's response to visual and auditory input

b. Describe how the infant responded to a bell or rattle

c. Describe how the infant responded to consultant/parent versus bell/rattle/red ball

d. Describe how the infant responded to auditory versus visual stimulation.

e. When the infant was awake, describe what helped the infant achieve, or maintain, an alert state. Describe what were the behaviors (or signs) that the infant could achieve or be maintained in an alert state.

5. Infant's Response to Cuddling

a. Describe the infant's response to being cuddled (In Arms, Upright on Shoulder)

b. Describe how the infant's responses were different or similar to 'In Arms' versus 'Upright on Shoulder'.

6. Infant's Signs of Stress (review signs of stress detailed in Lester and Tronick, 2004[23]

a. Describe the signs of stress the infant showed during the exam (indicate items on the Stress/Abstinence Scale items)

b. Describe what caused, or preceded the onset of these signs of stress

c. Describe how each stress sign was correlated with state and motor behavior.

d. Describe what followed the onset of each sign of stress: on the consultant/parent, and the infant's part.

7. Motor Behavior, Motor Movement and Motor Coordination.

a. Describe the infant's overall motor tone during the consult

b. Describe the infant's overall quality of movement.

c. Indicate the number of startles during the consult.

d. Describe significant reflex responses, those that were under or over responsive.

e. Describe the quality of sucking.

f. Describe how the infant's motor tone and motor control correlated with the infant's state and physiological responses.

8. Recommendations for Caretaking:

a. Based on the summary above, indicate recommendations for caregiving. See Boukydis, 2008[34] for examples. Also, use NNNS summary from NNNS manual Boukydis, Bigsby and Lester, 2004 [21].

[Base recommendations on the summary above. Be as specific as possible about the infant's behavior and the types of management responses or behavior needed]
